# One-Pot Microwave-Assisted Synthesis of Carbon Dots and *in vivo* and *in vitro* Antimicrobial Photodynamic Applications

**DOI:** 10.3389/fmicb.2021.662149

**Published:** 2021-06-21

**Authors:** María Paulina Romero, Fernanda Alves, Mirian Denise Stringasci, Hilde Harb Buzzá, Heloísa Ciol, Natalia Mayumi Inada, Vanderlei Salvador Bagnato

**Affiliations:** ^1^São Carlos Institute of Physics, University of São Paulo, São Carlos, Brazil; ^2^Departamento de Materiales, Facultad de Ingeniería Mecánica, Escuela Politécnica Nacional, Quito, Ecuador; ^3^Hagler Fellow, Texas A&M University, College Station, TX, United States

**Keywords:** carbon dot, photodynamic therapy, antibacterial photodynamic therapy (aPDT), carbon-based materials, carbon-based photosensitizer, antibacterial materials, *Staphylococcus aureus*, nanomedicine

## Abstract

Carbon-based photosensitizers are more attractive than the other ones based on their low cost, high stability, broadband of light absorption, tunable emission spectra, high quantum yield, water solubility, high resistance to metabolic degradation, and selective delivery. These properties allow multiple applications in the field of biology and medicine. The present study evaluated *in vitro* and *in vivo* the antimicrobial photodynamic effect of a one-pot microwave produced C-DOTS based on citric acid. The *in vitro* assays assessed the effectiveness of illuminated C-DOTS (C-DOTS + light) against *Staphylococcus aureus* suspension and biofilm. The concentrations of 6.9 and 13.8 mg/mL of C-DOTS and light doses of 20 and 40 J/cm^2^ were able to reduce significantly the microorganisms. Based on these parameters and results, the *in vivo* experiments were conducted in mice, evaluating this treatment on wounds contaminated with *S. aureus.* The viability test showed that C-DOTS–mediated photodynamic inactivation reduced 10^4^ log of the bacteria present on the skin lesions. These results, altogether, showed that antibacterial photodynamic therapy using C-DOTS is a promising and viable treatment for Gram-positive bacteria-infected wounds.

## Graphical Abstract

Brief description of the procedures carried out in this study. *In vivo* and *in vitro* antibacterial photodynamic therapy (aPDT) studies, where aPDT mediated by C-DOTS and blue LED light against *S. aureus* was evaluated.

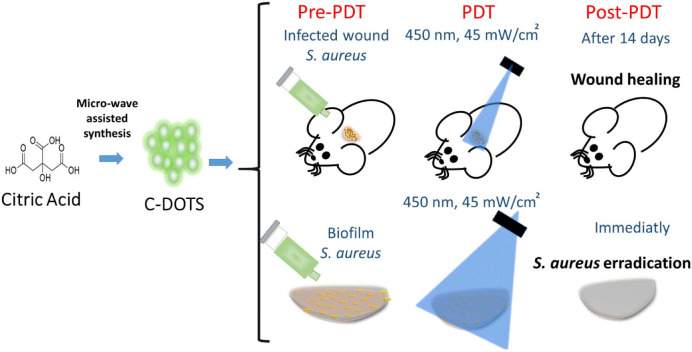

## Introduction

During the coronavirus disease 2019 (COVID-19) pandemic, 50% of the COVID-19 victims had a secondary bacterial (Chen et al., 2020) or fungal (Cox et al., 2020) infection. Coinfections also played an important role in other global pandemics. In the 1918 influenza outbreak, most of the deaths were due to a subsequent bacterial infection, mainly of them caused by *Streptococcus pneumonia.* In 2009, the failure results in the treatment of H1N1 influenza pandemic were also associated with bacterial coinfections (Resistencia a los antibióticos, 2020). Because of the importance of coinfections in the severity of global diseases and also associated with the emergence of bacterial resistance to antibiotics (Tablan et al., 2004; Gordon and Lowy, 2008), the search for new, efficient, and cheap mechanisms for bacterial control is needed.

*Staphylococcus aureus* is one of the most common and problematic bacteria that is able to develop antibiotic resistance. *S. aureus* is a Gram-positive bacterium commonly isolated from skin and oropharyngeal tract of healthy individuals and is responsible for superficial infections, pneumonia, and sepsis (Tablan et al., 2004; Gordon and Lowy, 2008). This bacterium has the ability to form biofilm, which is considered a high virulence factor that facilitates the infection in the host. Biofilms are a complex microbial community, where microorganisms produce an extracellular matrix (ECM) to protect them (Donlan, 2002; Donlan and Costerton, 2002).

The chemical composition and characteristics of Gram-positive bacteria, such as *S. aureus*, form a cationic ECM, which determines different relationships between the matrix and the environment. The matrix exhibits a highly hydrated structure with hydrophilic and hydrophobic groups. These characteristics protect the biofilm from dehydration and antimicrobial agents. Moreover, biofilms show a dynamic and complex architecture that is able to adapt according to environmental and internal changes. The presence of water channels provides nutrient and oxygen transportation through the biofilm and also enables cell-to-cell communication via quorum sensing molecules (Donlan, 2002; Donlan and Costerton, 2002). All these features make biofilms the hardest microbial organization to be inactivated by all types of antimicrobial drugs, playing an important role for disease control, and they are a challenge for healthcare professionals.

Nanotechnological approaches are a powerful tool for microbial control, mainly when they are associated with antibacterial photodynamic therapy (aPDT) and involve the use of carbon-based materials such as graphene, graphene oxide (GO), reduced GO, carbon quantum dot (C-DOTS), and graphene quantum dots (GQDs). Carbon-based materials are attractive for their low cost and high stability and emerged as a new class of broad−spectrum antimicrobial agents (Karahan et al., 2018).

Antibacterial photodynamic therapy has been proposed as a technique to treat clinical relevant infectious diseases, such as dental biofilms, ventilator-associated pneumonia, chronic wound infections, oral candidiasis, and chronic rhinosinusitis (Hu et al., 2018). It has been demonstrated that aPDT has a great potential for inactivating many classes of microorganisms, and it has the advantage of being minimally invasive and having low incidence of side effects, and it is suitable for rapid and repetitive application (Maisch, 2009; Yin et al., 2015; Cieplik et al., 2018).

Antimicrobial PDT involves the combination of a non-toxic photosensitizer and a light in an appropriate wavelength, which interact with the molecular oxygen-producing reactive oxygen species (ROS) that are able to selectively kill microbial cells. The ROS may include radical ions, such as superoxide (O_2_^⋅–^), hydroxyl radical (^⋅^OH), and/or singlet oxygen (^1^O_2_), and their production has been associated with the type I and/or type II photodynamic effects (Dolmans et al., 2003). *In vitro* and *in vivo* aPDT studies have demonstrated a substantial reduction of biofilms, in which the ROS are produced during photoactivation and attack adjacent targets, including proteins, lipids, and nucleic acids present within the biofilm matrix, on the cell surface and inside the microbial cells (Hu et al., 2018; Dong et al., 2020).

Carbon-based photosensitizers such as fullerene, GO, GQD, and graphitic carbon nitride have demonstrated low photosensitization efficiency, poor water solubility, or complex synthetic conditions, restricting their biological applications (Luo et al., 2014; Nie et al., 2020). On the other hand, C-DOTS presents effective photodynamic action, with the advantage of being non-toxic, photostable, and versatile and having high quantum yield (Dong et al., 2020).

Several authors have studied the antibacterial effects of C-DOTS, under blue and white light irradiation against different bacteria cells (Dong et al., 2018; Li et al., 2018; Marković et al., 2018; Travlou et al., 2018). Nie et al. (2020) synthesized CQDs based on citric acid and 1,5-diaminonaphthalene in relation 1:2. aPDT studies demonstrated good results of this treatment for the inactivation of Gram-negative (*Escherichia coli*) and Gram-positive (*S. aureus*) bacteria upon visible light illumination (λ ≥ 420 nm, 65 ± 5 mW/cm^2^; 60 min) (Nie et al., 2020). Sidhu et al. (2017) also synthesized C-DOTS based on citric acid with penicillin adsorbed or covalently attached to the C-DOTS. Authors demonstrated *in vitro* that both compounds under light irradiation exhibited activity against *S. aureus*, multidrug-resistant *E. coli*, *E. coli*, and methicillin-resistant *S. aureus* (Sidhu et al., 2017).

Other works have developed C-DOTS/photosensitizing nanocomposites (Du et al., 2016; Achadu and Nyokong, 2017; Nwahara et al., 2018) and C-DOTS/polymer (Marković et al., 2019; Bayoumy et al., 2020; Kováčová et al., 2020) with antibacterial activity. These studies have shown good results; however, there is no *in vitro and in vivo* C-DOTS study covering a very simple, cheap, and straightforward synthesis and its effective application as a photosensitizer in aPDT.

In general, C-DOTS are prepared via bottom-up syntheses using pyrolysis or carbonization of some organic precursors (citric acid, L-glutamic acid, and glucose) (Dong et al., 2012; Wu et al., 2013; Li et al., 2017). The carbonization of organic precursors through hydrothermal treatment has been used through the microwave-assisted and autoclave techniques, and they have been used in different biomedical applications. Besides that, their production occurs from abundant and inexpensive precursors, favoring their broad and low-cost application (Kumawat et al., 2017). The aim of the present work involved the simple one-pot hydrothermal synthesis, characterization of C-DOTS, and the evaluation of its aPDT effectiveness *in vitro* and *in vivo* against *S. aureus*. Only citric acid was used as the sole carbon source to prepare C-DOTS based on the preparation of C-DOTS via a standard microwave procedure, enabling its facile synthesis, being considered a cheap and green material with promising biomedical applications.

## Materials and Methods

### Synthesis and Characterization of C-DOTS

The water-soluble C-DOTS were prepared via microwave from the hydrothermal treatment of citric acid. First, 100 g of citric acid was dissolved 100 mL of deionized water in a glass beaker. Then, this solution was irradiated in a common microwave (Panasonic, Brazil) at high potency, for 8 min in an open atmosphere. The color of the liquid changed from colorless to pale yellow and strong yellow, indicating the formation of C-DOTS (Dong et al., 2012). Then, C-DOTS were purified by repeated dialysis in ultrapure water for 2 days (double dialyses bag molecular weight 1,300 Da). Finally, the solution was dried using a hot-air oven (Phoenix Luferco, Araraquara, Brazil) to produce solid structures of C-DOTS.

The C-DOTS sample was characterized by tapping-mode atomic force microscopy (Bruker Dimension Icon AFM) and transmission electron microscopy (TEM, JEOL–JEM2100 LaB6 HR operated at 200 kV). For TEM characterization, samples were prepared by dropping the C-DOTS solution on ultrathin carbon film-coated copper grid, 300 meshes. The luminescence emission measurements were performed at room temperature on a Cary Eclipse, Agilent technology spectrofluorometer, and a Varian Cary^®^ 50 UV-VIS system spectrophotometer.

Other techniques, including infrared spectroscopy [Agilent Technologies, Cary 630 Fourier transform infrared spectroscopy (FTIR)], Raman spectroscopy (Renishaw RM2000, laser HeNe, and 632.8-nm wavelength), and XRD spectroscopy (BRUKER APEX II Duo, two-copper, and molybdenum microsources, and low-temperature OXFORD system), were adopted to characterize the C-DOTS. In addition, the charge and size were evaluated by zeta potential and DLS measurements (Brookhaven Instruments Corporation MODELO: 90 plus particle size analyzer wavelength 659 nm).

### *In vitro* C-DOTS–mediated aPDT

#### Bacterial Suspension and Treatment

The *S. aureus* standard strain from the American Type Culture Collection (25923) was maintained in brain–heart infusion (BHI, Kasvi, São José dos Pinhais, Brazil) supplemented with glycerol (40%) at −20°C. The strain was reactivated by transferring 1 mL of the stock cultures to a tube containing 9 mL of BHI media and incubated at 37°C overnight. This culture was used to prepare bacterial suspension in phosphate-buffered saline (PBS). The suspension was standardized at an optical density of 0.2 arbitrary units (a.u.), determined in a spectrophotometer (Varian Cary^®^ 50 UV-Vis Spectrophotometer-Agilent, Santa Clara, CA, United States), which is equivalent to 10^8^ cells/mL. Then, 2 mL of bacterial suspension was transferred to a centrifuge tube for centrifugation [3,000 revolutions/min (rpm), 10 min]. The supernatant was removed, and 2 mL of C-DOTS solution with 0.345, 3.45, or 6.9 mg/mL diluted in PBS was added.

An aliquot of 0.5 mL of the resultant solution was transferred to a 24-well plate and incubated for 40 min at 37°C protected from light. Then, the plate was irradiated with blue light (450 nm; 40 mW/cm^2^) at the fluencies of 0, 20, 40, and 60 J/cm^2^, corresponding to irradiation times of 0, 8.3, 16.7, and 25 min, respectively. To determine cell survival, aliquots of the contents of each sample were serially diluted 10-fold in sterile saline. Aliquots of each serial dilution were plated in duplicate over the surfaces of BHI agar, and all plates were aerobically incubated at 37°C for 24 h. Then, the colony-forming units per milliliter (CFU/mL) were calculated. Control groups consisted of untreated bacteria suspension, the isolated application of light, and bacterial incubation with C-DOTS in the dark. Experiments were performed in five replicates for each experimental group on three different occasions.

#### Biofilm Formation and Treatment

The standardized bacterial suspension was prepared at the same conditions described previously, and it was used to form biofilm (10^8^ cells/mL) in PBS. For this, 1 mL of cell suspension was transferred to a 24-well plate and incubated at 37°C under agitation (75 rpm) for 90 min (adhesion phase). After the adhesion period, samples were carefully washed twice with 1 mL of saline to remove unbound cells and metabolites. Then, 1 mL of tryptic soy broth (TSB) was added in each well, and plates were maintained at 37°C in a shaker incubator (75 rpm) for 24 h. After this period, TSB culture medium was removed, and 1 mL of fresh medium was added. The plates were maintained at 37°C under agitation for a further 24 h, completing 48 h of biofilm formation (maturation stage). After this period, the content of each well was removed, and biofilms were washed twice with 1 mL of saline.

After the biofilm formation, 1 mL of PBS/C-DOTS solution at the concentrations of 3.45, 6.9, or 13.8 mg/L was added to the biofilms. Samples were incubated for 40 min at 37°C protected from light, and irradiated with blue light (450 nm; 40 mW/cm^2^) at the fluencies of 0, 20, 40, and 60 J/cm^2^, corresponding to irradiation times of 0, 8.3, 16.7, and 25 min, respectively.

After treatments, biofilms were detached by rubbing the pipette tip for 30 s on the bottom of the wells. Aliquots of the contents of each sample were serially diluted 10-fold in sterile saline. Duplicate 25-μL aliquots were spread over the surface of BHI agar plates. Control groups consisted of biofilms and light, biofilms and C-DOTS in the dark, and untreated biofilms.

All plates were aerobically incubated at 37°C for 24 h, and the CFU/mL values were calculated. Experiments were performed in five replicates for each experimental group on three different occasions.

### *In vivo* C-DOTS–Mediated aPDT

All experimental procedures were approved by the Institutional Animal Care and Use Committee (protocol 7658051218) of Sao Carlos Institute of Physics. The induction of skin wounds in Swiss mice was performed using the methodology proposed by Takakura et al. (2003) and Mima et al. (2010) with some modifications. The animals were immunosuppressed by two subcutaneous injections of prednisolone at a dose of 100 mg/kg of body weight using the medication methylprednisolone acetate 40 mg/mL. Immunosuppression was performed on the first and fifth days of the experiment.

On the second day of the experiment, the animals were anesthetized with an intraperitoneal injection of ketamine (30 mg/kg) and xylazine (13 mg/kg) solution in addition to diazepam injection at the dose of 5 mg/kg. The animals were trichotomized, and the wound was produced on the skin with a 3-mm-diameter punch. After wound procedure, 50 μL of bacterial suspension standardized at 108 cell/mL was added on the wound.

On the seventh day of the experiment and, therefore, after 6 days of bacterial inoculation, the animals were anesthetized with ketamine and xylazine solution (same concentration) and divided into four groups, with three animals in each group, as follows: control, only light, C-DOTS, and C-DOTS + light (aPDT). Mice of aPDT group received topical application of 50 μL of C-DOTS at a concentration of 13.8 mg/mL for 40 min in the dark and after incubation; LED illumination was performed with 60 J/cm^2^ (28 min) at the wavelength of 450 nm. Other animals received only the application of C-DOTs or light in the same parameters, and the control group received no treatment.

Immediately after applying the treatments, the microorganisms were collected using a sterile swab soaked in a saline solution that was scrubbed for 30 s over the wound. The collection was subjected to serial dilutions (10^0^–10^5^) and plated in duplicate on the BHI agar medium. Plates were incubated for 24 h at 37°C, and after this period, CFU/mL values were calculated. A second collection was also performed 7 days after the treatments, following the same procedure.

Colony-forming units per milliliter values were transformed into base-10 logarithms and were evaluated for the distribution and homogeneity of variance among the groups evaluated. One-way analysis of variance followed by the Tukey test was applied for multiple comparisons (*p* = 0.05).

## Results and Discussion

### Characterization of the C-DOTS

**FIGURE 1:**
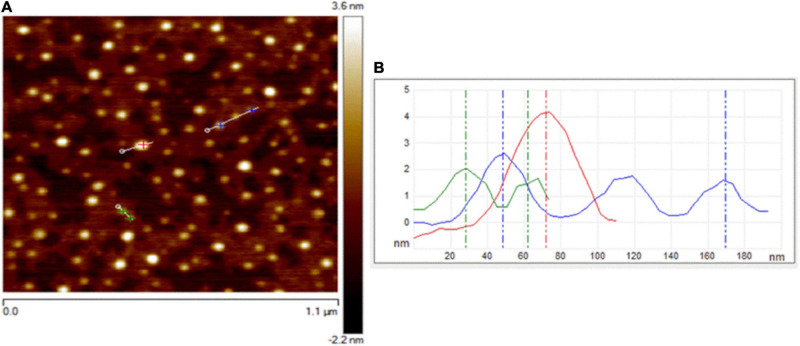
AFM images of C-DOTS. **(A)** AFM image of C-DOTS deposited on a mica substrate. **(B)** The height profiles along the lines of the panel from C-DOTS.

Atomic force microscopy and TEM were used to visualize the size, morphology, and structure of C-DOTS. The AFM images of C-DOTS shown in Figures 1A,B present the height profile along the small lines and indicate the thickness of the C-DOTS is in the range of 1.5–4.5 nm.

**FIGURE 2:**
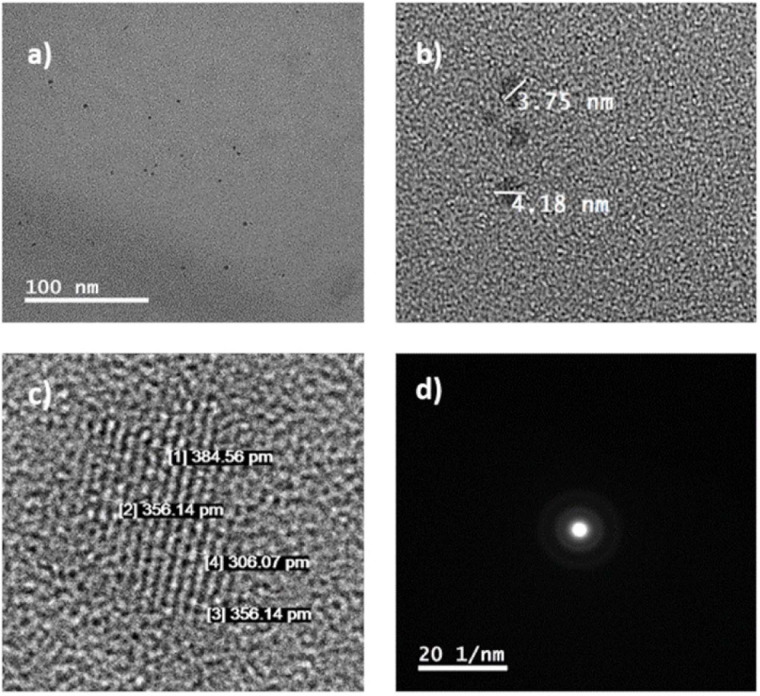
**(a,b)** C-DOTS TEM images. **(c,d)** High-resolution TEM images and corresponding 2D FFT image.

A TEM image of the C-DOTS is shown in Figure 2a. In Figure 2b, the size of C-DOTS is shown, which is equivalent to 3.75 and 4.18 nm. The mean diameter of the C-DOTS is 3.81 nm. The high-resolution TEM showed the lattice fringes with a lattice spacing around 0.35 ± 0.02 nm (Figure 2c), which corresponds to the lattice fringes of (0 0 2) planes (Zhou et al., 2007; Dong et al., 2013). In addition, the fast Fourier transform (FFT) pattern (Figure 2d) reveals that these structures are crystalline. Both AFM and TEM demonstrated that C-DOTS have circular shape with a diameter range of 1.5–4.5 nm and with a crystalline structure belonging to the carbon family. This result is comparable with the results found by Tang et al. (2016) and Van Tam et al. (2014).

**FIGURE 3:**
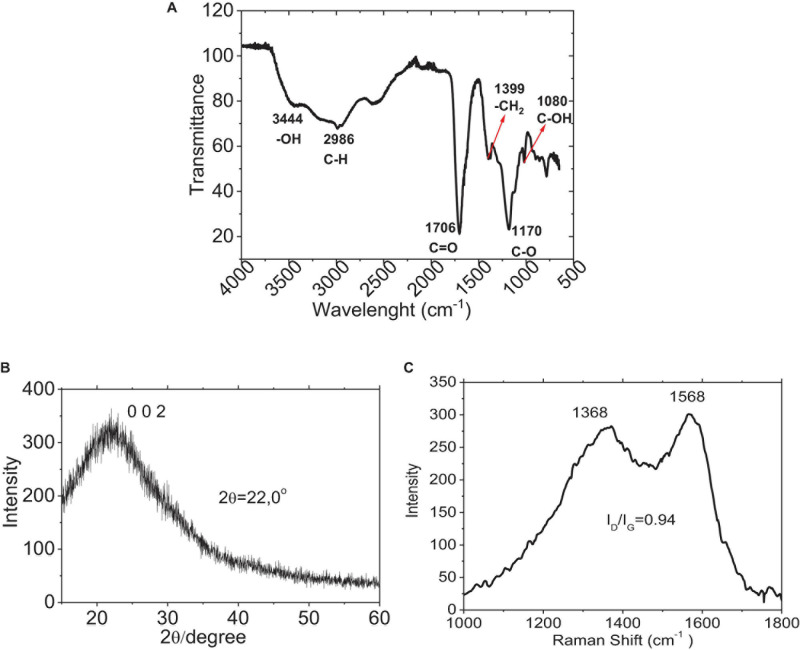
Characterization of C-DOTS. **(A)** FTIR spectrum of C-DOTS. **(B)** XRD pattern for the C-DOTS. **(C)** Raman spectrum of C-DOTS.

To investigate the difference in oxygen-related functional groups on C-DOTS, FTIR spectra (Figure 3A) were performed. The presence of the carbonyl group (C=O 1706 cm^–1^) and the hydroxyl group (–OH 3,444 cm^–1^) was observed, and C-DOTS showed absorption of stretching vibration C–H at 2,986 cm^–1^ and stretching vibration of –CH_2_ at 1,399 cm^–1^. Additionally, the C-DOTS showed absorption of stretching vibration C–O at 1,170 cm^–1^ and C–OH at 1,080 cm^–1^ (Kumawat et al., 2017). These results corroborate with those obtained by Dong et al. (2012), where GQDs and GO were synthesized based on the carbonization of citric acid. The analysis of FTIR spectrum suggested that GQDs contain incomplete carbonization of citric acid, compared to the GO, which exhibits complete carbonization of it.

The XRD pattern of C-DOTS (Figure 3B) revealed a single broad diffraction peak at 2θ = 26° that corresponded to the crystal lattice distance of (0 0 2) (*d*_002_ = 0.34 nm), and demonstrated a well-ordered lamellar structure with layered regularity (Dang et al., 2016).

The presence of oxygen-related functional groups on C-DOTS also creates disorders as defects and creates different hybridized atoms in the graphitic carbon skeleton, where both types of hybridized carbon atoms (sp_2_ and sp_3_) are prevalent. The XRD results are consistent with the Raman spectrum of C-DOTS. Figure 3C shows the Raman spectrum of C-DOTS, where the D-band (related to sp_3_ hybridized carbon atoms and the presence of structural defects) and the G-band (associated with sp_2_ hybridized carbon atoms) are characteristic of carbon-based material. The ID/IG ratio for C-DOTS is equivalent to 0.94, which indicates that the C-DOTS present high crystallinity and disorder in the skeleton of C-DOTS.

**FIGURE 4:**
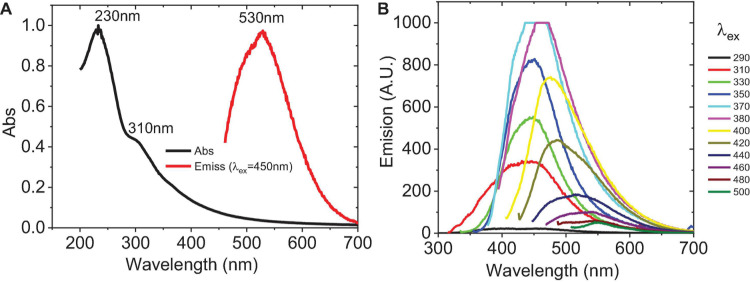
Optical properties of C-DOTS. **(A)** UV-VIS spectra (black line) and emission spectra (red line). λ_*ex*_ = 450 nm, λ_*em*_ = 530 nm. Images above: C-DOTS solution (yellow) without irradiation and C-DOTS emitting light green light when irradiated with 350-nm light. **(B)** Emission spectra of C-DOTS. Excitation wavelength from 290 to 500 nm (side frame) with their respective emission spectra (same color).

UV-VIS absorbance spectra and photoluminescence (PL) emission were obtained. Figure 4A indicates that C-DOTS have an absorption band at λ < 240 nm (characteristic of the π–π^∗^ transitions of aromatic bonds C=C) and another smaller band around 310 nm, which belongs to the n–π^∗^ transition of C=O or the COOH group (Liu and Kim, 2015; Tang et al., 2016). The PL spectra are generally broad and dependent on the excitation wavelength (Shen et al., 2011). Figure 4B shows that C-DOTS have the highest luminescence in the visible part of the spectrum, with the maximum emission intensity approximately at 450 nm (λ_*ex*_ = 370 nm), then it is reduced and shifted at higher wavelengths to approximately 548 nm (λ_*ex*_ = 500 nm), and it is consistent with previous fluorescence analysis (Li et al., 2010; Pan et al., 2010; Ristic et al., 2014).

### Stability Analysis of C-DOTS

**FIGURE 5:**
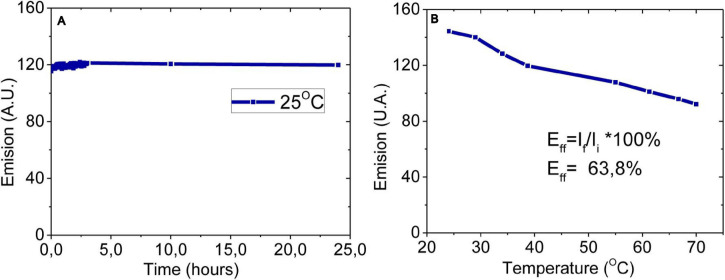
Stability analysis of C-DOTS. **(A)** Twenty-four hours of photoirradiation at constant temperature (25°C). **(B)** Photoirradiation based on temperature, from 25 to 70°C. λex = 450 nm, λem = 530 nm, pH 5.5.

Stability is the main characteristic of the C-DOTS that depends on the nature of the environment in which they are exposed. Poor stability leads to aggregation and deterioration of lattice attributed to a disturbance of fluorescence properties (Sidhu et al., 2017). To investigate the stabilities of the C-DOTS by photo-oxidation and PL properties, they were examined over 24 h in the presence of blue light (Xenon lamp of Cary Eclipse, Agilent technology spectrofluorometer) with λ_*exc*_ = 450 nm and λ_*emis*_ = 530 nm (Figure 5A). It was shown that in the presence of light, a very small decrease in the fluorescence intensity was observed, and it is consistent with previous stability analysis (Wang et al., 2017; Dager et al., 2019).

The stability of C-DOTS when the temperature was increased is shown in Figure 5B. It was observed that, increasing the temperature, there is a decrease in the fluorescence emission. Surprisingly, at 70°C, the fluorescence intensity of the C-DOTS decreased only 36.20%. Unlike amorphous C-DOTS, the crystalline structure of the C-DOTS synthesized in the present study could explain their excellent photostability (Liu et al., 2017; Nie et al., 2020). The C-DOTS solution in water remains without any precipitation, and its fluorescence emission intensity was maintained after 90 days, keeping it in the dark.

## Photodynamic Effects

### C-DOTS-Mediated aPDT in the Inactivation of Planktonic and Biofilm Cultures of *S. aureus*

Biofilm is a microbial community embedded in a self-produced extracellular polymeric matrix (ECM) attached to a biological or non-biological surface. Compared to planktonic cells, biofilm exhibits specific physiological and metabolic states (Hu et al., 2018). According to Karunakaran et al. (2011), the biofilm is formed with three elements: the ECM, the microbial cells, and the intracellular biomolecules (Karunakaran et al., 2011). During aPDT-mediated biofilm inactivation, oxidative damage occurs from extracellular polymeric substances (EPS) to intracellular biomolecules (Barra et al., 2015).

**FIGURE 6:**
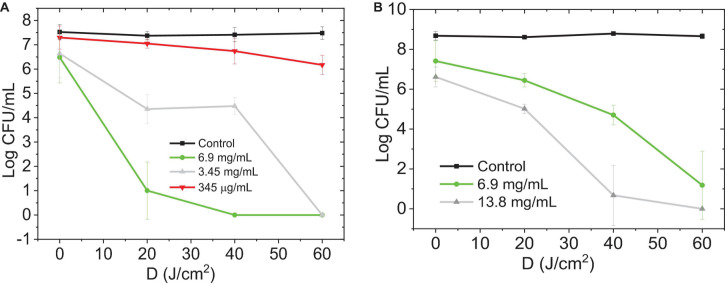
Photoexcited C-DOTS reduce the number of bacterial colonies (log CFU/mL) based on the light dose 450 nm, 40 mW/cm^2^ delivered. **(A)** Planktonic *S. aureus* + C-DOTS. **(B)** Biofilm of *S. aureus* + C-DOTS. The “control” group refers to the bacteria + light system. The group “0 J/cm^2^” corresponds to the bacteria + C-DOTS system, where it was observed that the C-DOTS by themselves decreased the bacterial population in 10^2^ CFU/mL.

The colony count method demonstrated a significant reduction in the number of CFU/mL in *S. aureus* in both planktonic and biofilm cultures exposed to C-DOTS photoexcited by light at 450 nm and 40 mW/cm^2^ (Figure 6). The decrease in log (CFU/mL) can be observed as a function of the dose of blue light delivered to the planktonic culture (0, 20, 40, and 60 J/cm^2^) and the C-DOTS concentration (345 μg/mL, 3.45 mg/mL, 6.9 and 13.8 mg/mL). As expected at higher C-DOTS concentrations, the log (CFU/mL) reduction was greater, and when the concentration of 6.9 mg/mL was applied, the total elimination of *S. aureus* suspension was achieved. For the biofilm cultures (Figure 6B), twice of the C-DOTS concentration was used (13.8 mg/mL) compared to the planktonic culture, in order to obtain complete elimination of the *S. aureus* bacteria. For both systems mentioned previously, the complete elimination of the *S. aureus* bacteria was obtained using the light dose of 40 J/cm^2^.

The dose–response curve is a relationship among the viability reduction of planktonic and biofilm cultures, C-DOTS concentrations, and the light delivery of 450 nm 40 mW/cm^2^. Because of the variability among the bacteria cells, the dose–response curve has a sigmoid shape (Romero et al., 2020). The threshold dose shows the distribution formed by the response of the bacterial to C-DOTS and light around a maximum dose of light, Dp. This curve was obtained by deriving the dose–response curve.

**FIGURE 7:**
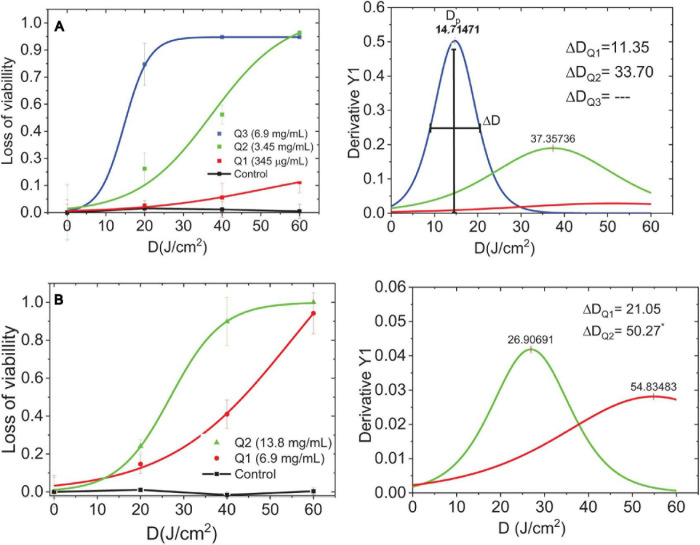
Dose-response curves and threshold dose distribution. **(A)** Planktonic *S. aureus* + C-DOTS. **(B)** Biofilm of *S. aureus* + C-DOTS. Dp is the maximum dose of delivery light, ΔD [full-width half-maximum (FWHM)] is the threshold dose distribution curves obtained from the dose–response curves. *The FWHM value found of the complete curve.

The dose–response curves and their corresponding threshold dose distribution of *S. aureus* planktonic (Figure 7A) and biofilm (Figure 7B) were evaluated. These curves were obtained from the results of colony counting of planktonic and biofilm of *S. aureus* in the presence of C-DOTS as a direct killing effect. The fitting of the inverse of standard colony counting (loss of the viability) curve was performed in the Origin8 program using a sigmoidal curve.

The loss of viability distribution was characterized by the width. Figure 7A is showing a measure at half maximum (full-width half-maximum) and their peak center, which corresponds to the maximal dose amplitude. de Faria et al. (2018) argued that quantifying tolerance in a population of bacteria is a measure of how heterogeneous the population of bacteria is with respect to the threshold dose. This variability is related to the tolerance (de Faria et al., 2018).

Parameter is summarized in Supplementary Figure 1A and is shown as a function of the C-DOTS concentration, where the higher the concentration of C-DOTS, the lower the value. This means that a small dose of light is required to eliminate a great amount of bacterial population. This behavior was observed in planktonic and biofilm cultures. For the analysis of the parameter, the concentration of 345 μg/mL in the planktonic culture was eliminated as it was an incomplete curve where its respective value was not distinguished.

The highest value corresponds to the biofilm *S. aureus* + C-DOTS; this indicates that the biofilm has the strongest resistance condition in relation to the planktonic culture. The planktonic culture is the most sensitive for both concentrations of C-DOTS. It is interesting to note that when doubling the concentration of C-DOTS for both cultures (Supplementary Figure 1B), the value is reduced 2.5 times for planktonic form and 2 times for the biofilm. This means that with higher concentrations in both cultures, the difference in sensitivity is significant. In summary, the planktonic culture showed a better response to C-DOTS concentration compared to the biofilm. All control groups had the same order of magnitude (1 × 10^8^ CFU/mL) from the initial standardized suspensions.

Planktonic culture needs C-DOTS concentrations lower than 6.9 mg/mL to achieve total bacterial killing, whereas concentrations greater than it were required to the biofilm. Biofilm can tolerate antibiotic levels 10–1,000 times higher than their planktonic counterparts (Ceri et al., 1999). The biofilm living form is a protective lifestyle that allows pathogenic bacteria and fungi to survive in hostile environments (Hall-Stoodley et al., 2004). In the clinical area, biofilms lead to infections that are difficult to eradicate, as microbes are protected from the attack of the host defense system and exhibit antimicrobial resistance (Hu et al., 2018). For this reason, the results obtained in the present study are of great importance, as aPDT with C-DOTS was able to inactivate even biofilm communities.

The relative variability of ΔD/Dp is shown in Supplementary Figure 2A, and in both cases, ΔD/Dp decreased with the increase in C-DOTS concentration; in higher concentrations of C-DOTS, there was a more homogeneous response of *S. aureus* bacteria to 450 nm irradiation (40 mW/cm^2^ LED light), with a minimal variability of response. In both cultures, plankton and biofilm, the response to the presence of C-DOTS and blue light presented similar values of ΔD/Dp for low and high concentrations (Supplementary Figure 2B), concluding that both cultures have a similar response to C-DOTS–mediated aPDT.

### C-DOTS-Mediated aPDT Zeta Potential and DLS

To confirm the electrostatic interactions, the zeta potentials of bacterial cells were evaluated after treatments. The zeta potential of C-DOTS and *S. aureus* and *S. aureus* + C-DOTS were −22.09 and −31.60 mV, respectively, but the zeta potential of *S. aureus* + C-DOTS + light was −15.33 and 4.5 mV as shown in Supplementary Figures 3A–D. It was shown that the zeta potential of the bacteria may be attributed to the cell surface components of Gram-positive bacteria, in the case of *S. aureus* (Ran et al., 2019).

These results indicate that the photodynamic effect produced a charge change in the bacteria + C-DOTS + light solution, tending to be positive. The hydrodynamic sizes of the *S. aureus*, *S. aureus* + C-DOTS, and *S. aureus* + C-DOTS + light was 966.2, 1,018.6, and 1,064 nm, respectively (Supplementary Figure 3E). A small difference was observed between maximum sizes of the studied solutions, but a greater distribution of sizes was observed for the bacteria + C-DOTS + light, with larger sizes than the bacteria + C-DOTS. These values lead to the conclusion that in the illumination to obtain the photodynamic effect (elimination of bacteria), there is formation of aggregates in the solution.

Ran et al. (2019) synthetized carbon dots prepared by the solvothermal treatment of dimethyloctadecyl[3-(trimethoxysilyl)propy]ammonium chloride (Si-QAC CDs) and quaternized carbon dots without long alkyl chains (termed TTPAC CDs). The zeta potentials of *S. aureus* bacteria after the treatment with TTPAC CDs and Si-QAC CD-treated (incubated for 2 h) indicated a higher binding affinity between the Si-QAC CDs and the bacterial surfaces. Especially, the similar zeta potentials of the bacterial cells at the CD concentrations of 30 and 50 μg/mL suggested that the *S. aureus* bacteria were well coated by the CDs at a concentration of 30 μg/mL or higher (Ran et al., 2019).

From the results obtained in the present study, due to the presence of light, the zeta potential increased, as observed by Ran et al. (2019). It suggests that the presence of light in the *S. aureus* + C-DOTS solution allows a greater binding affinity between C-DOTS and bacterial surfaces, allowing total elimination of bacteria observed in Figure 6A.

The C-DOTS concentrations evaluated in the present study were between 0 and 6.9 mg/mL, and compared to the concentrations of various types of C-DOTS used by other authors (between 0 and 1 mg/mL) (Yang et al., 2016, Yang et al., 2019; Ran et al., 2019), the concentration of C-DOTS used in this study was approximately seven times higher. This could be explained by the traces of citric acid in which they were duly purified in the stage of the synthesis of C-DOTS. In *in vitro* studies developed in HDFn fibroblasts cell line (Supplementary Information 1), it is observed that at a concentration of 6.9 mg/mL, cell survival decreases to approximately 40% for a light dose of 63 J/cm^2^ and 450-nm irradiation. Concluding that at concentrations greater than and equal to 6.9 mg/mL, cellular cytotoxicity is already observed under the effect of aPDT. This effect was observed using 38 and 63 J/cm^2^ of light doses, after 4 and 24 h of aPDT.

### *In vivo* C-DOTS Antimicrobial Activity

The use of C-DOTS as an antimicrobial agent *in vivo* was investigated with and without light. The “control” group was performed to analyze the natural processes of infection healing in wound contaminated with *S. aureus*. The “light” group was performed to confirm that the light parameters used did not cause any damage in the contaminated wound.

**FIGURE 8:**
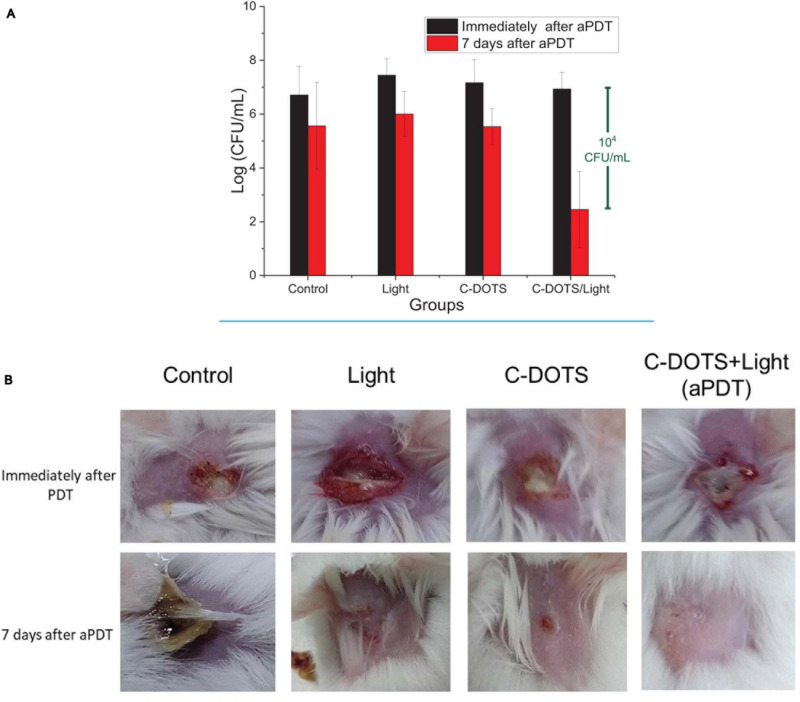
*In vivo* aPDT. **(A)** Log (CFU/mL) of *S. aureus* bacteria in mouse immediately aPDT (black blocks) and 7 days after the procedures (red blocks) in control, light, C-DOTS, and C-DOTS + light (aPDT) groups (*n* = 3). **(B)** Photographs of mice wounds infected with *S. aureus*. Same groups as panel **(A)** (*n* = 3). The C-DOTS concentration was 6.9 mg/mL; the wound was infected with 10^8^ cells/mL of bacteria suspension; the LED light was applied with the dose of 60 J/cm^2^ (28 min) at 450 nm.

Bacteria were collected from the wound immediately and 7 days after all procedures to obtain the effectiveness of each group. Immediately after the procedures, there was no great difference among the groups (*p* ≥ 0.05), as shown in Figure 8A, where all groups presented colony counts in the same order of magnitude (1 × 10^7^ CFU/mL). However, on day seven post-treatment, a significant reduction of the viability was observed in the C-DOTS + light group. The control groups (no treatment, light, and C-DOTS) showed 1 × 10^6^ CFU/mL, whereas the C-DOTS + light group showed in the order of 10^2^ CFU/mL, resulting in a reduction of 10^4^ log CFU/mL (Figure 8A). A small reduction was observed in the “control” (only bacteria in lesion), “light” (bacteria + light), and “C-DOTS” (bacteria + C-DOTS in dark) groups. This reduction may be attributed to immune system of the animals that combated the infection by itself.

The induced infection on the skin of mice also showed different features after 7 days of the procedures. The lesions in the control group had a large crust with a very purulent aspect. The light and C-DOTS groups exhibited small wounds (Figure 8B). However, a complete response and good healing were observed in the C-DOTS + light group, as shown in Figure 8B. The skin of all mice was completely recovered after 21 days (with and without treatment). When evaluating the cytotoxic effects of aPDT in HDFn fibroblasts cell line (Supplementary Information 1), it was observed that at the concentration of 6.9 mg/mL, there is a cell survival of approximately 35%; this survival is reduced by increasing the concentration of C-DOTS. However, it was demonstrated that *in vivo* the C-DOTS concentration used (6.9 mg/mL) had no visible effect (Figure 8B).

The antimicrobial mechanism of C-DOTS–mediated aPDT was caused by the production of ROS, which leads to oxidative stress as described by Marković et al. (2019) and Zhang et al. (2018). The generation and clearance of ROS in bacterial cells are balanced in normal conditions; however, the excessive production of ROS favors the oxidation of the cells. This unbalanced state produces oxidative stress, which damages the components of bacteria. The mechanisms of action include the adhesion of C-DOTS to the bacterial surface, the photoinduced production of ROS, the disruption and penetration of the bacterial cell wall/membrane, and the induction of oxidative stress with damages to DNA/RNA, leading to alterations or inhibitions of important gene expression and oxidative damages to proteins and other intracellular biomolecules (Al Awak et al., 2017; Li et al., 2018).

Some articles have described that photoactivate C-DOTS and other nanoparticles can transfer electron or energy to oxygen or biomolecules generating cytotoxic-free radicals and ROS (Ipe et al., 2005; Yaghini et al., 2009). Besides the death of microorganisms, there is some evidence that aPDT can stimulate the host immune system, which contributes to the healing process, as observed in the better aspect of lesions 7 days after C-DOTS + light treatment (Castano et al., 2006).

Antibacterial photodynamic therapy using traditional PS is often limited by its high cost, mainly in developing and/or underdeveloped countries. With the results obtained in this article, we are proposing C-DOTS as nanomaterials whose synthesis is cheap, easy, and green (there is no generation of toxic waste). These advantages make C-DOTS attractive to be used as photosensitizer in aPDT for antibacterial control.

## Conclusion

In summary, we developed C-DOTS from citric acid through a facile microwave-assisted synthesis, and it was evaluated in association with blue light to mediate aPDT *in vitro* and *in vivo* against *S. aureus*. The C-DOTS aqueous solutions exhibited strong fluorescence under 450-nm light and fluorescence emission stability for 24 h of continuous irradiation and also after 90 days of being stored in the dark. The *in vitro* study demonstrated that C-DOTS–mediated aPDT was able to eliminate *S. aureus* suspension and biofilm. The *in vivo* study was conducted based on the *in vitro* study. Seven days after the aPDT procedure on the mice contaminated wounds, a 10^4^ log of reduction in the *S. aureus* population compared to the controls and complete healing of the tissue were observed. The use of C-DOTS as a photosensitizer in aPDT proved to be promising in the treatment of biofilms and infected wounds, making these water-soluble C-DOTS a simple, cheap, and efficient agent for aPDT both for *in vitro* and *in vivo* studies.

## Data Availability Statement

The original contributions presented in the study are included in the article/Supplementary Material, further inquiries can be directed to the corresponding author.

## Ethics Statement

The animal study was reviewed and approved by CEUA N 7658051218.

## Author Contributions

MR: synthesis of carbon dots, development of *in vitro* studies in *S. aureus* bacterial cells, and manuscript writing. FA: development of studies in biofilms and English language review. MS and HB: development of *in vivo* studies (mice) and English language review. HC: development of *in vitro* studies on epithelial cells. NI and VB: guide in the conception, design of the work, analysis, and interpretation of data. All authors contributed to the article and approved the submitted version.

## Conflict of Interest

The authors declare that the research was conducted in the absence of any commercial or financial relationships that could be construed as a potential conflict of interest.
