# Supplementary material for: One-Pot Microwave-Assisted Synthesis of Carbon Dots and in vivo and in vitro Antimicrobial Photodynamic Applications
Source: Front Microbiol. 2021 Jun 21;12:662149. doi: 10.3389/fmicb.2021.662149 (PMC8255795; doi:10.3389/fmicb.2021.662149)
Supplement: Supplementary file 1 [file Data_Sheet_1.pdf]

Support Information

One-pot Microwave Assisted Synthesis of Carbon Dots and *in vivo* and *in vitro* Antimicrobial Photodynamic Applications

María Paulina Romero[1,2,], Fernanda Alves[1], Mirian Denise Stringasci[1], Hilde Harb Buzzá[1], Heloísa Ciol[1], Natalia Mayumi Inada[1] and Vanderlei Salvador Bagnato[1,3]*

Figure S1. *Threshold dose parameters. a) DP and b) table summary*

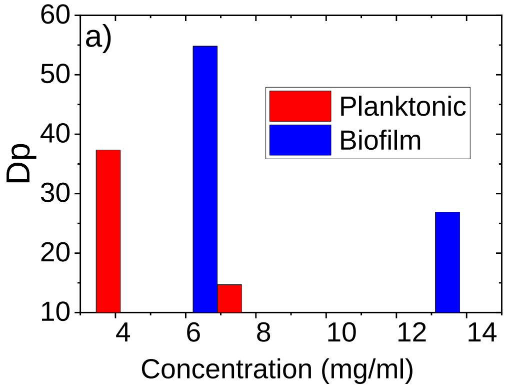

b)

	Concentration [mg/mL]	*Dp*
Planktonic	3.45	37.35
	6.9	14.71
Biofilm	6.9	54.83
	12.8	26.91

Figure S2. *ΔD/DP ratio. a) Planktonic+C-DOTS (red) and Biofilm+C-DOTS (blue). b) summarized data*

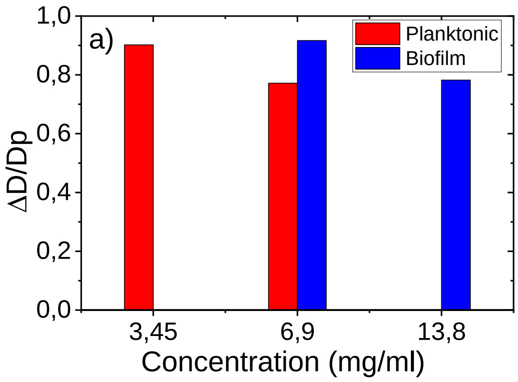

b)

	C-DOTS Low	*C-DOTS High*
Planktonic	0.90	0.77
Biofilm	0.92	0.78

Figure S3*. Zeta Potential* a) C-DOTS, b) *S. aureus*, c) *S. aureus*+C-DOTS, d) *S. aureus*+C-DOTS+light 60 J/cm^2 and 6.4 mg/mL and *e*) Hydrodynamic diameter obtained from DLS measurements.

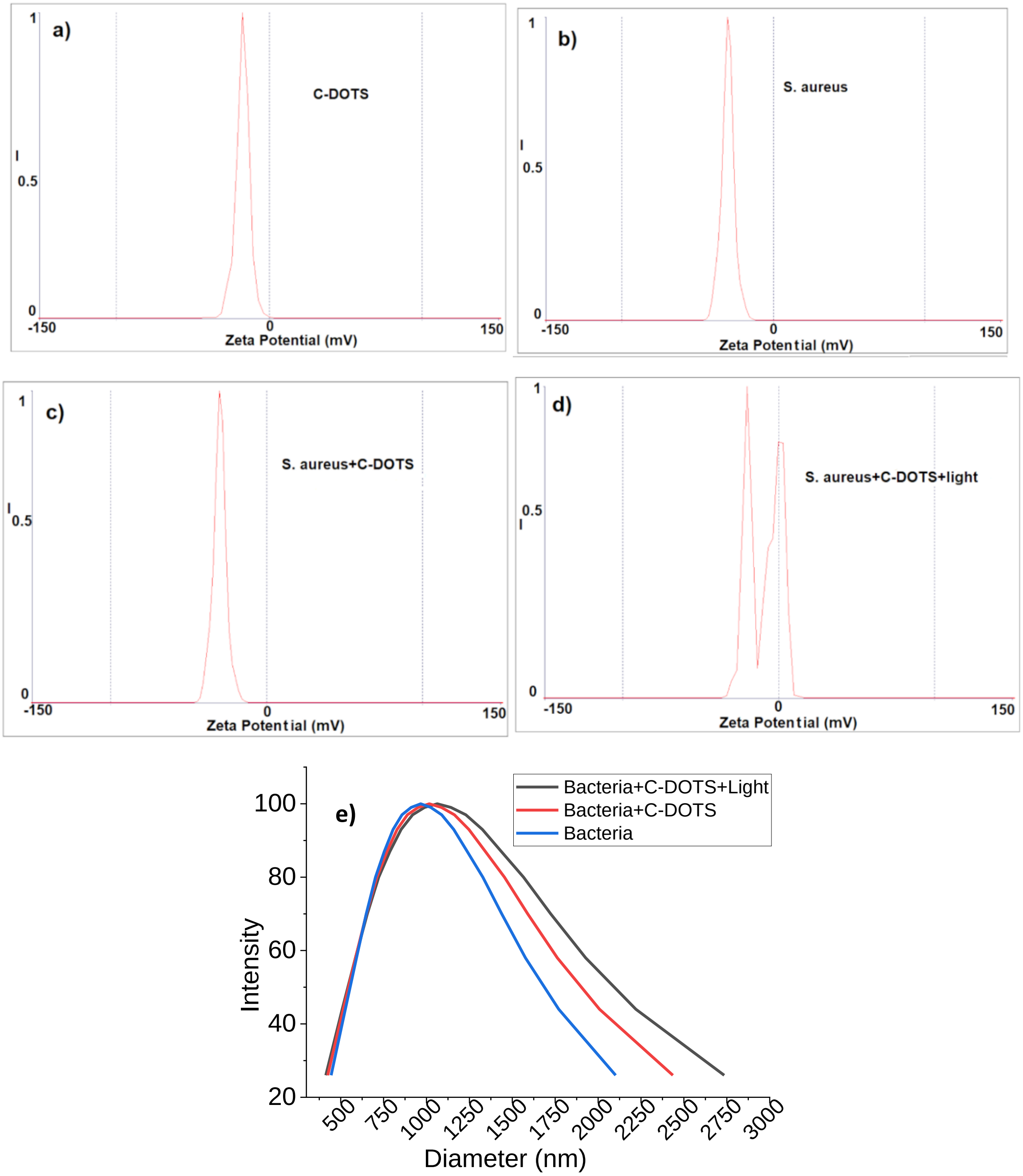

Information S1

In vitro cytotoxicity of C-DOTS in fibroblasts

The effect of aPDT on HDFn fibroblasts was evaluated. To this end, the cytotoxic effects of C-DOTS in the presence of HDFn fibroblasts was studied. Figure 1a shows the aPDT effect on HDFn fibroblasts for 38 and 63 J/cm^2 in the presence of various concentrations of C-DOTS. It is noted that at a C-DOTS concentration of 3.5 mg/mL there is 80% cell survival, but at 7.5 mg/mL survival decreases to approximately 30%.

Concentrations equal or lower than 3.75 mg/mL did not result in higher cell death rates. The same behavior is observed for the control sample (not aPDT), suggesting that cell death was caused by C-DOTS exposure. Incubation time 45 min was chosen based on the photodynamic inactivation protocol used in the in vivo study.

The cytotoxic effects of aPDT were analyzed with an incubation time of 4h and 24h where the same behavior is observed for the two incubation times (Fig. 1b). It is observed that at the concentration of 3.75 mg/mL, the survival of the cells falls by around 35% and at the concentration of 7.5 mg/mL, no cells survive. Ge et al. (2016) and Lan et al. (2018) showed fully cell uptake of C-DOTS after 4 and 24 h of incubation and toxicity effects after these periods[i(a,b)]. In the present study, a variation in behavior is observed between the concentrations of 1.88 mg/mL and 7.5 mg/mL for these long incubation periods.

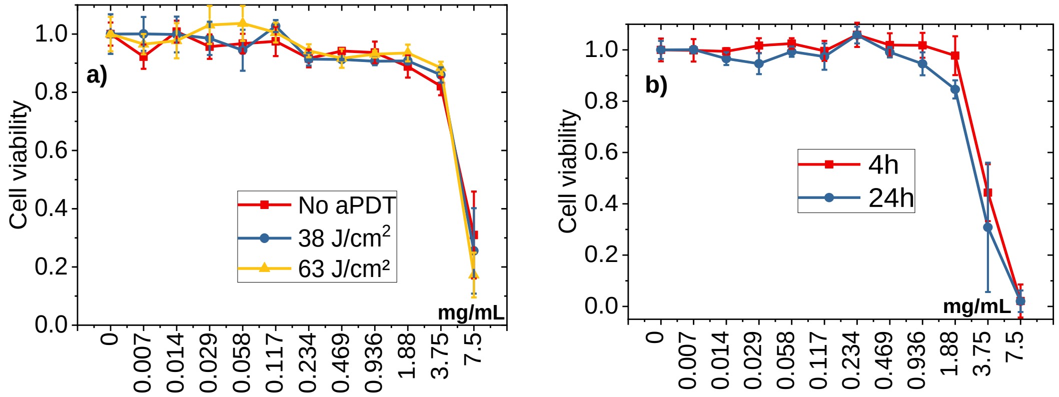

Fig. 1 Cell viability of HDFn fibroblasts a) Evaluation of the effect of aPDT for doses of 38 and 63 J / cm2 in HDFn fibroblasts (cell viability after incubation of HDFn cells with C-DOTS for 45 minutes, followed by irradiation). b) Evaluation of the effect of aPDT for incubation times of 4 and 24 hours (cells incubation time with C-DOTS were 4h or 24h). Relative cells death was determined based on control samples (0 µg/mL).

The cytotoxicity of C-DOTS in cells was studied by several authors. Ge J et al. (2016) demonstrated in vitro no toxicity of C-DOTS (from polythiophene benzoic acid as carbon source) to B16-F60 skin cancer cells, where 100% of the B16-F60 cells remained alive after 4h of dark incubation with C-DOTS, even when C-DOTS concentrations were higher than 200 µg/mL. When PDT was applied using laser irradiation at 635 nm and 0.1 W/cm^2, 40% of cells were destroyed after incubation with 200 µg/mL[ii] . Lan et al. (2018) studied the biocompatibility and phototoxicity of C-DOTS (using 1,3,6-trinitropyrene and Na2SO3 as the precursors) to HeLa cells. Authors observed that the cell viability was near 100% after incubation with C-DOTS at concentrations ranging from 12.5 to 100 µg/mL for 24 h in dark condition. Even when the concentration of C-DOTs increased to 200 µg/mL, the cell viability

was over 80%. When HeLa cells were exposed to 800 nm laser femtosecond pulsed laser irradiation, only about 10% of cells were viable, suggesting the excellent biocompatibility and high phototoxicity of the C-DOTS[iii]. Ge et al. (2014) assessed the photodynamic activity of C-DOTS (from polythiophene (PT2) as carbon source) in HeLa cells. Cells were irradiated with light at 635 nm in the presence of C-DOTS from 0.036 to 1.8 mM. Authors observed that when using 0.036 mM of GQDs, the cell viability was equivalent to 60%. The viability decreased when higher C-DOTS concentrations were applied (equivalent to 20% for the 1.8 mM GQD solution). Authors also observed that C-DOTS have little effect on the survival of HeLa cells in the dark even at the concentration of 1.8 mM, indicating low cytotoxicity and good biocompatibility of C-DOTS[iv]. The studies mentioned are consistent with the results obtained in the present study, which also shows that at low concentrations (<1.88 mg/mL) there is no significant cytotoxicity effect at both light doses, 38 and 63 J/cm^2.

The antibacterial mechanism of C-DOTS-mediated PDT in cells is caused by the presence of ROS. Ge J. et al (2014) evaluated the morphological changes caused by PDT in HeLa cells due to ROS generation, and they observed that this treatment lead to the shrinkage of cells, the formation of numerous bubbles and photo-induced cell death was followed by nuclear condensation[v].

---

[i] a) Ge J, Jia Q, Liu W, et al. Carbon Dots with Intrinsic Theranostic Properties for Bioimaging, Red-Light-Triggered Photodynamic/Photothermal Simultaneous Therapy In Vitro and In Vivo. *Adv Healthc Mater*. 2016;5(6):665-675. doi:10.1002/adhm.201500720. b) Lan, M., Guo, L., Zhao, S., Zhang, Z., Jia, Q., Yan, L., Xia, J., Zhang, H., Wang, P. and Zhang, W. (2018), Carbon Dots as Multifunctional Phototheranostic Agents for Photoacoustic/Fluorescence Imaging and Photothermal/Photodynamic Synergistic Cancer Therapy. Adv. Therap., 1: 1800077. doi:10.1002/adtp.201800077

[ii] Ge J, Jia Q, Liu W, et al. Carbon Dots with Intrinsic Theranostic Properties for Bioimaging, Red-Light-Triggered Photodynamic/Photothermal Simultaneous Therapy In Vitro and In Vivo. *Adv Healthc Mater*. 2016;5(6):665-675. doi:10.1002/adhm.201500720.

[iii] Lan, M., Guo, L., Zhao, S., Zhang, Z., Jia, Q., Yan, L., Xia, J., Zhang, H., Wang, P. and Zhang, W. (2018), Carbon Dots as Multifunctional Phototheranostic Agents for Photoacoustic/Fluorescence Imaging and Photothermal/Photodynamic Synergistic Cancer Therapy. Adv. Therap., 1: 1800077. doi:10.1002/adtp.201800077

[iv] Ge, J., Lan, M., Zhou, B., Liu, W., Guo, L., Wang, H., Jia, Q., Niu, G., Huang, X., Zhou, H., Meng, X., Wang, P., Lee, C., Zhang, W., Han, X. A graphene quantum dot photodynamic therapy agent with high singlet oxygen generation. Nature Communications 2014; 5 (1): 1-8. **doi: 10.1038/ncomms5596**

[v] Ge, J., Lan, M., Zhou, B., Liu, W., Guo, L., Wang, H., Jia, Q., Niu, G., Huang, X., Zhou, H., Meng, X., Wang, P., Lee, C., Zhang, W., Han, X. A graphene quantum dot photodynamic therapy agent with high singlet oxygen generation. Nature Communications 2014; 5 (1): 1-8. **doi: 10.1038/ncomms5596**
